# Research Topics of the Bioinformatics of Gene Regulation

**DOI:** 10.3390/ijms24108774

**Published:** 2023-05-15

**Authors:** Yuriy L. Orlov, Anastasia A. Anashkina, Vadim V. Kumeiko, Ming Chen, Nikolay A. Kolchanov

**Affiliations:** 1 The Digital Health Institute, I.M. Sechenov First Moscow State Medical University of the Ministry of Health of the Russian Federation (Sechenov University), 119991 Moscow, Russia; 2 Institute of Cytology and Genetics, Siberian Branch of the Russian Academy of Sciences, 630090 Novosibirsk, Russia; kol@bionet.nsc.ru; 3 Life Sciences Department, Novosibirsk State University, 630090 Novosibirsk, Russia; 4 Agrarian and Technological Institute, Peoples’ Friendship University of Russia, 117198 Moscow, Russia; 5 Engelhardt Institute of Molecular Biology, Russian Academy of Sciences, 119991 Moscow, Russia; 6 Institute of Life Sciences and Biomedicine, Far Eastern Federal University, 690922 Vladivostok, Russia; vkumeiko@yandex.ru; 7 Department of Bioinformatics, College of Life Sciences, Zhejiang University, Hangzhou 310058, China

The study of gene expression regulation raises the challenge of developing bioinformatics tools and algorithms, demanding data integration. A recent journal Special Issue, “Bioinformatics of Gene Regulations and Structure—2022”, collected papers on bioinformatics applications originally based on the materials presented at “Bioinformatics of Genome Regulation and Structure/Systems Biology” (BGRS\SB-2022) in July 2022 in Novosibirsk, Russia (https://bgrssb.icgbio.ru/2022, accessed on 10 May 2023). BGRS is the biannual bioinformatics conference series, and has taken place in Novosibirsk since 1998. It facilitates a broad scientific discussion of systems biology and genomics achievements, published in thematic journal issues [1,2,3]. Based on these conference discussions, we can review current trends in medical genomics [4] and cancer genomics [5]. Gene expression regulation at a transcriptional level was a key problem discussed at the conference series and in *IJMS* Special Issues [5,6,7].

The molecular mechanisms of human disease progression [4], as well as gene expressions in laboratory animal and plant models [2], have been being studied using omics technologies. The presented analytical techniques were discussed at the BGRS meeting series in Novosibirsk, Russia [1,2,3], highlighting recent advances in the fields of evolution, biomedicine, and biotechnology, and have been reviewed in existing (https://www.mdpi.com/journal/ijms/special_issues/Bioinformatics_Gene, accessed on 10 May 2023) and new *IJMS* issues (https://www.mdpi.com/journal/ijms/special_issues/MVA479KFR7, accessed on 10 May 2023). In fact, there are ten thematic paper collections (Special Issues) on bioinformatics, gene expression regulation, computational genomics, and computational biology in MDPI journal issues referring to past BGRS series conferences [1,8].

This collection builds on studies in the field of gene expression initially presented in *Frontiers in Genetics* [4] and *BioMed Central* Special Issues [1], and also in *International Journal of Molecular Sciences* [2,3,5]. The examination of this topic has also continued through thematic issues in *Genes* [6] (www.mdpi.com/journal/genes/special_issues/Transcriptional_Regulation_Tumor, accessed on 10 May 2023) and *Life* [7] (https://www.mdpi.com/journal/life/special_issues/computational_genomics_life, accessed on 10 May 2023). Here, we have collected papers with an overarching theme of bioinformatics of gene expression regulation, initially considering biomedical applications [2,5].

The issue “Bioinformatics of Gene Regulations and Structure—2022” showcased insights into the fields of genomics, transcriptomics, and proteomics, as well as the works conducted in model organisms. This Special Issue contains nine research manuscripts and one review, each concerning a bioinformatics solution or tool for the analysis of the molecular mechanisms of gene expression regulation.

The papers present novel bioinformatics models on gene expression regulation and chromatin immunoprecipitation-sequencing (ChIP-seq) technology to analyze transcription factor binding to DNA, gene regulation at transcription and translation levels, single cell sequencing tools, and applications in model organisms.

We open this collection of papers with works discussing the molecular mechanisms of DNA binding regulating gene transcription. Anastasia Melikhova et al. [9] discussed evolutionary constraints on the DNA double helix in RNA Pol II core promoters. This classical approach used representative sets of aligned gene promoter sequences of fifteen eukaryotic species. The evolutionarily stable and heterogeneous secondary structure of Pol II core promoters was revealed, including the TATA-box [10]. It should be noted that the TATA-box structure and the Polymerase II binding have been described in detail, along with the development of the specialized tools, in recent *IJMS* publications in the “Bioinformatics of Gene Expression” Special Issue by M.P.Ponomarenko and colleagues [11,12] in relation to the same conference series.

Gene expression is regulated at transcription and translation levels [10,13]. Bioinformatics methods for assessing the efficiency of different stages of gene expression, including translation elongation, were presented by Aleksandra Korenskaia et al. [14]. The authors estimated mRNA sequence features, such as codon usage bias and mRNA secondary structure properties, and evaluated correlation coefficients between experimentally measured protein abundance and predicted elongation efficiency characteristics for the set of prokaryotes belonging to diverse taxonomic groups. The study extends the results on elongation efficiency estimates presented earlier [15].

Gene expression is controlled at the nucleotide and structural levels, as well [16]. The next group of papers discussed applications of the chromosomal regulation of gene expression in animal models. Artem Nurislamov and co-authors [17] used a lampbrush chromosomes model to analyze DNA methylation in chicken. The authors performed a single-cell methylome analysis of chicken diplotene oocytes. A. Nurislamov and colleagues characterized methylation patterns in these cells, obtained methylation-based chicken genome segmentation, and identified oocyte-specific methylated gene promoters. The role of chromatin architecture alterations in genomes has been discussed in our topical journal issue by the same science group by V. Fishman [18]. In the next study, Evelyn Kabirova et al. [19] reviewed distal gene expression regulation in animals considering the evolution of the loop extrusion machinery. Loop extrusion machinery consists of various proteins with different time origins [20]. The evolutionarily conserved core of all extrusion complexes is formed by SMC (structural maintenance of chromosomes) proteins. The review by Kabirova and co-authors [19] covers the roles of SMC complexes in gene regulation, DNA repair, and chromatin topology.

Evgenia Solodneva et al. [21] considered genetic models in cattle. The authors analyzed the genetic structures of transboundary and local cattle based on STR (short tandem repeat) markers. To determine the population genetic characteristics and clarify the phylogenetic relationships of modern representatives of hundreds of cattle populations from different regions of the world, the authors analyzed a large set of STR data (more than 10 individuals) including unique native cattle populations and breeds. The classification extends the results presented recently by this science group [22]. 

The next group of papers showed new bioinformatics tools for transcription regulatory region analysis [23]. ChIP-seq technology provides abundant data for the annotation of regulatory modules and nucleotide motifs in gene promoter regions [10,24]. Victor Levitsky et al. [23] presented a new web-server Web-MCOT (Motifs Co-Occurrence Tool) for ChIP-seq data analysis aimed for use in nucleotide motifs co-occurrence searching. Note that, in the paper by the same science group highlighted in an *IJMS* Special Issue [25], V. Levitsky and colleagues presented a study on the cooperative binding of protein transcription factors to DNA as the mechanism of transcription regulation. Transcription factor binding and the clustering of the binding sites based on ChIP-seq have been studied in plant [26] and yeast genomes [27]. However, the technology of bulk sequencing moving to single cell sequencing and its variants challenges the development of new tools [28]. In this Special Issue, Mikhail Raevskiy and colleagues [29] presented the Epi-Impute software for single-cell RNA-seq (scRNA-seq) and ATAC-seq analysis. scRNA-seq data contain many dropouts, hampering downstream analyses due to the inefficient capture of mRNAs in individual cells [30]. The method suggested is intended for dropout imputation by reconciling expression and epigenomic data. Epi-Impute leverages single-cell ATAC-seq data to reduce the number of dropouts [29]. The work by the same group by Y. Medvedeva on scRNA-seq data analysis application was published in an *IJMS* issue on medical bioinformatics [31].

Finally, a group of papers presents text mining and machine learning applications in gene regulation studies. Vladimir Ivanisenko et al. [32] present a new version of the ANDDigest (Associative Network Design—Digest) tool with improved AI-based short names recognition of genes. This tool, as an extension of the ANDSystem (Associative Network Discovery System) [33,34] for the text mining of science literature and network reconstruction, has been successfully applied to a range of biomedical problems [35] discussed in *IJMS* Special Issues (https://www.mdpi.com/journal/ijms/special_issues/Medical_Genetics_2022, accessed on 10 May 2023). Currently, it is employed in applications for model plant organisms [36]. Aleksandar Veljkovic and colleagues [37] developed a new tool, BioGraph, for querying diverse biological metadata. The presented model enables information retrieval using interlinked entities from highly diverse biological datasets in a unified manner, extending the capabilities of the ANDDigest tool [32,33].

Zeping Cai et al. [38] presented the genome-wide mining of the tandem duplicated type III polyketide synthases and in *Senna tora* crop. The authors analyzed the tandem duplicated genes *S. tora*. The study [38] will provide helpful information for the further functional analysis of the CHS-L genes in the regulation of anthraquinone biosynthesis in *S. tora* [39].

The problems associated with plant bioinformatics and discovering molecular mechanisms of gene expression in plant models are presented in a parallel Special Issue, “Plant Biology and Biotechnology: Focus on Genomics and Bioinformatics 2.0” (https://www.mdpi.com/journal/ijms/special_issues/PlantBi_Biology, accessed on 10 May 2023). It can be noted that the next PlantGen-2023 conference, “Plant Genetics, Genomics, Bioinformatics and Biotechnology” will take place in Kazan, Russia, in July 2023 (https://plantgen2023.ofr.su/, accessed on 10 May 2023). The PlantGen conference series originated from the Institute of Cytology and Genetics SB RAS events, and developed in parallel to the BGRS conference series on bioinformatics. We collected bioinformatics papers on previous topical issues [2], as well. 

Thus, the current Special Issue series on bioinformatics has confirmed the research interest in gene expression regulation studies [2,3,5]. We can summarize the research topic papers’ content as covering regulatory region sequence analysis, ChIP-seq studies, and epigenetic and single cell sequencing applications. The model genomes studies also vary from human and animal genomes to recently annotated plant genomes.

The guest editors are happy to announce the Special Issue topic “New Sights into Bioinformatics of Gene Regulations and Structure” (https://www.mdpi.com/journal/ijms/special_issues/MVA479KFR7, accessed on 10 May 2023) of MDPI’s *IJMS*, as well as the next conference on system biology and bioinformatics in Russia in 2023 (https://conf.icgbio.ru/sblai2023/, accessed on 10 May 2023), considering Artificial Intelligence applications in bioinformatics. We also note the recent VII Congress of Russian Biophysicists in Krasnodar, Russia (http://rusbiophysics.ru/db/conf.pl?cid=1&lang=en&div=1, accessed on 10 May 2023), and the range of biophysical models related to molecular mechanisms of gene expression presented there. We hope that readers find these materials to be interesting and stimulating, and we will continue to collect papers on gene expression regulation for new *IJMS* journal issues.

## References

[1] Orlov Y.L., Baranova A.V., Hofestädt R., Kolchanov N.A. (2016). Computational genomics at BGRS\SB-2016: Introductory note. BMC Genom..

[2] Orlov Y.L., Tatarinova T.V., Anashkina A.A. (2021). Bioinformatics Applications to Reveal Molecular Mechanisms of Gene Expression Regulation in Model Organisms. Int. J. Mol. Sci..

[3] Orlov Y.L., Anashkina A.A., Klimontov V.V., Baranova A.V. (2021). Medical Genetics, Genomics and Bioinformatics Aid in Understanding Molecular Mechanisms of Human Diseases. Int. J. Mol. Sci..

[4] Orlov Y.L., Chen W.L., Sekacheva M.I., Cai G., Li H. (2022). Editorial: High-Throughput Sequencing-Based Investigation of Chronic Disease Markers and Mechanisms. Front. Genet..

[5] Anashkina A.A., Leberfarb E.Y., Orlov Y.L. (2021). Recent Trends in Cancer Genomics and Bioinformatics Tools Development. Int. J. Mol. Sci..

[6] Voropaeva E.N., Pospelova T.I., Orlov Y.L., Churkina M.I., Berezina O.V., Gurazheva A.A., Ageeva T.A., Seregina O.B., Maksimov V.N. (2022). The Methylation of the p53 Targets the Genes MIR-203, MIR-129-2, MIR-34A and MIR-34B/C in the Tumor Tissue of Diffuse Large B-Cell Lymphoma. Genes.

[7] Orlov Y.L., Anashkina A.A. (2021). Life: Computational Genomics Applications in Life Sciences. Life.

[8] Ignatieva E.V., Matrosova E.A. (2021). Disease-associated genetic variants in the regulatory regions of human genes: Mechanisms of action on transcription and genomic resources for dissecting these mechanisms. Vavilovskii Zhurnal Genet. Selektsii.

[9] Melikhova A., Anashkina A., Il’icheva I. (2022). Evolutionary Invariant of the Structure of DNA Double Helix in RNAP II Core Promoters. Int. J. Mol. Sci..

[10] Tsukanov A.V., Levitsky V.G., Merkulova T.I. (2021). Application of alternative *de novo* motif recognition models for analysis of structural heterogeneity of transcription factor binding sites: A case study of FOXA2 binding sites. Vavilovskii Zhurnal Genet. Selektsii.

[11] Rasskazov D., Chadaeva I., Sharypova E., Zolotareva K., Khandaev B., Ponomarenko P., Podkolodnyy N., Tverdokhleb N., Vishnevsky O., Bogomolov A. (2022). Plant_SNP_TATA_Z-Tester: A Web Service That Unequivocally Estimates the Impact of Proximal Promoter Mutations on Plant Gene Expression. Int. J. Mol Sci..

[12] Vishnevsky O.V., Chadaeva I.V., Sharypova E.B., Khandaev B.M., Zolotareva K.A., Kazachek A.V., Ponomarenko P.M., Podkolodny N.L., Rasskazov D.A., Bogomolov A.G. (2022). Promoters of genes encoding β-amylase, albumin and globulin in food plants have weaker affinity for TATA-binding protein as compared to non-food plants: In silico analysis. Vavilovskii Zhurnal Genet. Selektsii.

[13] Hui S., Silverman J.M., Chen S.S., Erickson D.W., Basan M., Wang J., Hwa T., Williamson J.R. (2015). Quantitative proteomic analysis reveals a simple strategy of global resource allocation in bacteria. Mol. Syst. Biol..

[14] Korenskaia A., Matushkin Y., Lashin S., Klimenko A. (2022). Bioinformatic Assessment of Factors Affecting the Correlation between Protein Abundance and Elongation Efficiency in Prokaryotes. Int. J. Mol. Sci..

[15] Sokolov V., Zuraev B., Lashin S., Matushkin Y. (2015). Web application for automatic prediction of gene translation elongation efficiency. J. Integr. Bioinform..

[16] Greenberg M.V.C., Bourc’his D. (2019). The diverse roles of DNA methylation in mammalian development and disease. Nat. Rev. Mol. Cell Biol..

[17] Nurislamov A., Lagunov T., Gridina M., Krasikova A., Fishman V. (2022). Single-Cell DNA Methylation Analysis of Chicken Lampbrush Chromosomes. Int. J. Mol. Sci..

[18] Belokopytova P., Fishman V. (2021). Predicting Genome Architecture: Challenges and Solutions. Front Genet..

[19] Kabirova E., Nurislamov A., Shadskiy A., Smirnov A., Popov A., Salnikov P., Battulin N., Fishman V. (2023). Function and Evolution of the Loop Extrusion Machinery in Animals. Int. J. Mol. Sci..

[20] Fudenberg G., Imakaev M., Lu C., Goloborodko A., Abdennur N., Mirny L.A. (2016). Formation of Chromosomal Domains by Loop Extrusion. Cell Rep..

[21] Solodneva E., Svishcheva G., Smolnikov R., Bazhenov S., Konorov E., Mukhina V., Stolpovsky Y. (2023). Genetic Structure Analysis of 155 Transboundary and Local Populations of Cattle (*Bos taurus, Bos indicus* and *Bos grunniens*) Based on STR Markers. Int. J. Mol. Sci..

[22] Svishcheva G., Babayan O., Lkhasaranov B., Tsendsuren A., Abdurasulov A., Stolpovsky Y. (2020). Microsatellite Diversity and Phylogenetic Relationships among East Eurasian Bos taurus Breeds with an Emphasis on Rare and Ancient Local Cattle. Animals.

[23] Levitsky V., Mukhin A., Oshchepkov D., Zemlyanskaya E., Lashin S. (2022). Web-MCOT Server for Motif Co-Occurrence Search in ChIP-Seq Data. Int. J. Mol. Sci..

[24] Euskirchen G.M., Rozowsky J.S., Wei C.L., Lee W.H., Zhang Z.D., Hartman S., Emanuelsson O., Stolc V., Weissman S., Gerstein M.B. (2007). Mapping of transcription factor binding regions in mammalian cells by ChIP: Comparison of array- and sequencing-based technologies. Genome Res..

[25] Levitsky V., Oshchepkov D., Zemlyanskaya E., Merkulova T. (2020). Asymmetric Conservation within Pairs of Co-Occurred Motifs Mediates Weak Direct Binding of Transcription Factors in ChIP-Seq Data. Int. J. Mol. Sci..

[26] Dergilev A.I., Orlova N.G., Dobrovolskaya O.B., Orlov Y.L. (2022). Statistical estimates of multiple transcription factors binding in the model plant genomes based on ChIP-seq data. J. Integr. Bioinform..

[27] Goh W.S., Orlov Y., Li J., Clarke N.D. (2010). Blurring of High-Resolution Data Shows that the Effect of Intrinsic Nucleosome Occupancy on Transcription Factor Binding is Mostly Regional, Not Local. PLoS Comput. Biol..

[28] Lähnemann D., Köster J., Szczurek E., McCarthy D.J., Hicks S.C., Robinson M.D., Vallejos C.A., Campbell K.R., Beerenwinkel N., Mahfouz A. (2020). Eleven grand challenges in single-cell data science. Genome Biol..

[29] Raevskiy M., Yanvarev V., Jung S., Del Sol A., Medvedeva Y.A. (2023). Epi-Impute: Single-Cell RNA-seq Imputation via Integration with Single-Cell ATAC-seq. Int. J. Mol. Sci..

[30] Xu J., Cui L., Zhuang J., Meng Y., Bing P., He B., Tian G., Pui C.K., Wu T., Wang B. (2022). Evaluating the performance of dropout imputation and clustering methods for single-cell RNA sequencing data. Comput. Biol. Med..

[31] Shevtsov A., Raevskiy M., Stupnikov A., Medvedeva Y. (2023). In Silico Drug Repurposing in Multiple Sclerosis Using scRNA-Seq Data. Int. J. Mol. Sci..

[32] Ivanisenko T., Demenkov P., Kolchanov N., Ivanisenko V. (2022). The New Version of the ANDDigest Tool with Improved AI-Based Short Names Recognition. Int. J. Mol. Sci..

[33] Ivanisenko T.V., Saik O.V., Demenkov P.S., Ivanisenko N.V., Savostianov A.N., Ivanisenko V.A. (2020). ANDDigest: A new web-based module of ANDSystem for the search of knowledge in the scientific literature. BMC Bioinform..

[34] Ivanisenko V.A., Saik O.V., Ivanisenko N.V., Tiys E.S., Ivanisenko T.V., Demenkov P.S., Kolchanov N.A. (2015). ANDSystem: An Associative Network Discovery System for automated literature mining in the field of biology. BMC Syst. Biol..

[35] Saik O.V., Klimontov V.V. (2022). Gene Networks of Hyperglycemia, Diabetic Complications, and Human Proteins Targeted by SARS-CoV-2: What Is the Molecular Basis for Comorbidity?. Int. J. Mol. Sci..

[36] Demenkov P.S., Oshchepkova E.A., Demenkov P.S., Ivanisenko T.V., Ivanisenko V.A. (2021). Prioritization of biological processes based on the reconstruction and analysis of associative gene networks describing the response of plants to adverse environmental factors. Vavilovskii Zhurnal Genet. Selektsii.

[37] Veljković A.N., Orlov Y.L., Mitić N.S. (2023). BioGraph: Data Model for Linking and Querying Diverse Biological Metadata. Int. J. Mol. Sci..

[38] Cai Z., Zhao X., Zhou C., Fang T., Liu G., Luo J. (2023). Genome-Wide Mining of the Tandem Duplicated Type III Polyketide Synthases and Their Expression, Structure Analysis of *Senna tora*. Int. J. Mol. Sci..

[39] Kang S.H., Lee W.H., Lee C.M., Sim J.S., Won S.Y., Han S.R., Kwon S.J., Kim J.S., Kim C.K., Oh T.J. (2020). De novo transcriptome sequence of Senna tora provides insights into anthraquinone biosynthesis. PLoS ONE.

